# Coverage Probability Analysis and Relay Placement Optimization for Two-Hop LoRa Networks with Random Traffic Activation

**DOI:** 10.3390/s26134156

**Published:** 2026-07-01

**Authors:** Zongliang Xu, Guicai Yu

**Affiliations:** 1 School of Intelligent Science and Engineering, Qinghai Minzu University, Xining 810000, China; 17671410397@163.com; 2 National Demonstration Center for Experimental Teaching of Communication Engineering, Qinghai Minzu University, Xining 810007, China

**Keywords:** LoRa, two-hop communication, random traffic activation, coverage probability, intra-SF and inter-SF interference, relay placement optimization

## Abstract

In LoRa uplink communication, direct edge-node-to-gateway transmission is affected by path loss, thermal noise, small-scale fading, and intra-spreading-factor (intra-SF) and inter-spreading-factor (inter-SF) interference under random traffic activation. These factors reduce the signal-to-interference-plus-noise ratio (SINR) and degrade coverage reliability. To address these issues, this study proposes an integrated framework for coverage probability analysis and relay placement optimization in two-hop LoRa networks with random traffic activation. First, a two-hop LoRa uplink network model is established, consisting of edge source nodes, a decode-and-forward (DF) relay, a central gateway, and potential interfering nodes. By incorporating distance-dependent path loss, receiver-side thermal noise power, and small-scale fading gains, a unified received-power model is formulated for the desired and interfering links. Second, a Bernoulli traffic-activation indicator is assigned to each potential interfering node to characterize its random transmission state and link the traffic activation probability, the active-interferer set, and the expected number of active interferers. An interference model is then developed to quantify the effect of random traffic activation on aggregate interference over communication links. To account for multi-spreading-factor (multi-SF) coexistence in LoRa, intra-SF interference and residual inter-SF interference are incorporated into the link-level SINR criterion, and the corresponding single-hop coverage probability is derived. Finally, based on the DF relaying protocol, successful end-to-end transmission is modeled as the joint event of successful first-hop decoding and successful second-hop forwarding, and a two-hop coverage probability model is constructed. A system-level coverage probability model is also developed to capture the complementarity between direct transmission and two-hop relaying. A relay placement optimization problem is then formulated to maximize the weighted average system coverage probability across multiple traffic states. The performance of fixed, random, and optimized relay placement schemes is compared. Simulation results demonstrate that the proposed random-traffic-aware relay placement optimization method substantially improves the end-to-end coverage probability compared with fixed and random relay placement schemes, thereby enhancing the communication reliability of edge-node transmissions.

## 1. Introduction

Low-power wide-area networks (LPWANs) have been widely adopted in agricultural monitoring, the Industrial Internet of Things (IIoT), and other IoT scenarios owing to their wide-area coverage and low power consumption [1]. As a representative LPWAN technology, LoRa enables long-range communication with low transmit power by employing chirp spread spectrum (CSS) modulation and a multi-spreading-factor (multi-SF) transmission mechanism [2,3]. Different spreading factors (SFs) provide different trade-offs among receiver sensitivity, transmission rate, and link coverage capability. Terminal nodes located farther from the gateway are typically assigned higher SFs and higher transmit power to improve edge-node transmission reliability [4,5]. The coverage performance of LoRa networks is affected not only by path loss, thermal noise, and small-scale fading but also by intra-SF interference, residual inter-SF interference arising from imperfect SF orthogonality, and random traffic activation [6,7].

In LoRa uplink transmission, edge nodes are typically located near the boundary of the gateway coverage area, resulting in long propagation distances to the gateway. Consequently, the received signal power is substantially attenuated by path loss and shadow fading, while thermal noise further degrades the link quality [8,9]. Compared with near-gateway links, edge-node links have a more constrained link budget and are therefore more likely to have a signal-to-noise ratio (SNR) or signal-to-interference-plus-noise ratio (SINR) below the demodulation threshold under identical noise and interference conditions [10]. Increasing the spreading factor or transmit power can improve the reliability of edge-node links to some extent. However, a higher SF reduces the data rate and prolongs the time-on-air (ToA), thereby increasing the probability of intra-SF collisions and the level of residual inter-SF interference [11,12,13]. LoRa uplink transmissions generally lack fine-grained power control and real-time inter-node coordination mechanisms, making it difficult for edge nodes to adapt their channel-access behavior in response to instantaneous interference conditions [14]. The communication reliability of edge nodes determines the effective coverage boundary of LoRa networks and is a key factor limiting further improvements in overall network coverage [15]. To address this issue, a relay node is introduced to assist in forwarding data from edge nodes, thereby dividing the long-distance direct link into two shorter hops. By reducing the per-hop propagation loss, this approach improves the end-to-end coverage probability.

A decode-and-forward (DF) relay can divide the long-distance source-to-gateway link into two shorter links, namely the source-to-relay and relay-to-gateway links, thereby mitigating the severe path loss encountered in direct edge-node-to-gateway communication [16,17]. In a DF relaying system, successful end-to-end transmission can be modeled as the joint event of successful first-hop decoding at the relay and successful second-hop forwarding to the gateway. If first-hop decoding fails, no valid decoded packet is available for second-hop forwarding [18]. Similarly, if the gateway fails to decode the second-hop transmission due to interference or noise, successful end-to-end transmission cannot be achieved. LoRa terminals typically adopt an ALOHA-based random access mechanism. Under this mechanism, the traffic load is time-varying and may be unsaturated. Therefore, the activation states of potential interfering nodes vary across observation cycles [19,20,21]. Changes in the traffic activation probability directly determine the number of active interfering nodes in each observation cycle. As a result, the SINR at the relay and gateway receivers varies with the traffic activation state [22].

LoRa multi-hop and relay-assisted architectures can extend network coverage and improve the connectivity of remote nodes. However, their performance is highly dependent on the relay transmission mode and relay placement strategy [23,24]. Random access and traffic activation alter the number of active interfering nodes, thereby affecting the link success probability through intra-SF collisions and residual inter-SF interference. Therefore, coverage probability analysis of two-hop LoRa networks should jointly account for the sequential transmission constraint imposed by two-hop relaying and the randomness of traffic-induced interference [25].

Existing studies addressing limited edge-node communication reliability have mainly focused on two directions: link-parameter optimization and relay-assisted transmission. Link-parameter optimization methods can improve edge-node link budgets by adjusting the spreading factor (SF), transmit power, or access parameters. However, such improvements involve inherent trade-offs: higher transmit power may increase the interference imposed on other nodes, whereas a higher SF improves receiver sensitivity at the cost of prolonged ToA and reduced system capacity [26]. Link-parameter optimization alone is insufficient to jointly balance edge-coverage enhancement, network-level interference control, and efficient spectrum utilization. In relay-assisted transmission, a relay node is introduced between the source node and the gateway, thereby reducing the per-hop transmission distance and improving edge-node link reliability. In a two-hop LoRa network, relay placement should not be treated as an isolated design variable; rather, it simultaneously affects the source-to-relay and relay-to-gateway link distances, the distances from interfering nodes to the relay and gateway receivers, and the SF-dependent interference structure across the two hops. Therefore, it is necessary to develop a two-hop coverage probability model under random traffic activation conditions and to optimize relay placement based on this model, thereby improving edge-node end-to-end transmission reliability.

Nguyen et al. [27] addressed the coverage limitation of edge nodes by developing a coverage probability model for two-hop LoRa networks. They derived analytical expressions for the coverage probability under multiple fading channel models and investigated the effects of intra-SF interference, inter-SF interference, and their joint impact on coverage performance. Relay placement was further optimized to improve edge-node coverage. However, the traffic activation process in their work was mainly characterized by a fixed activation probability or a deterministic number of active nodes, without explicitly modeling the relationship between traffic-load fluctuations and the active interferer set. Consequently, under varying traffic intensity, the coupling among traffic-induced interference, coverage probability, and optimal relay placement remains insufficiently characterized. Xu et al. [28] proposed a two-hop opportunistic amplify-and-forward (AF) relaying system for LoRa networks, in which the best relay was selected to reduce the bit error rate (BER) and improve the coverage probability. However, the additional forwarding time slot required by AF relaying reduces the throughput compared with conventional direct LoRa transmission. Moreover, their model primarily focuses on SNR analysis under Nakagami-m fading, while the coupling among relay placement and traffic-activation-induced variations in the active interferer set, intra-SF and inter-SF interference structures remains insufficiently characterized. Mahjoub et al. [29] investigated a best-relay selection scheme for two-hop DF relay-assisted LoRa networks. Their method selects the relay with the best link quality from a candidate relay set to improve the end-to-end transmission success probability and edge-node communication reliability. However, their study primarily focuses on the diversity gain obtained from candidate-relay selection rather than on the effect of relay placement itself on the coverage probability under random traffic activation and coupled interference conditions. Therefore, the coupling among traffic activation intensity, the active interferer set, the SF-dependent interference structure, and spatial relay placement remains insufficiently explored.

In summary, existing studies have sought to improve LoRa edge-node communication reliability from multiple perspectives, including two-hop coverage probability analysis, AF/DF relaying, and best-relay selection. However, several common limitations remain: (1) The traffic activation process is often represented by a fixed activation probability or a deterministic number of active nodes, thereby limiting the ability of such models to characterize the impact of random traffic-intensity variations on the resulting interference state. (2) Some studies primarily focus on fading-channel characterization or link-level signal-to-noise ratio (SNR) analysis, whereas the coupling between intra-SF interference and residual inter-SF interference remains insufficiently characterized. (3) Existing relay optimization methods primarily focus on relay placement or best-relay selection in static scenarios, whereas the dynamic variation of the optimal relay placement under random traffic-intensity variations remains insufficiently analyzed. To address these limitations, this study develops a coverage probability model for two-hop LoRa networks under random traffic activation. The proposed model jointly accounts for traffic-activation-induced interference variations, SF-dependent interference characteristics, path loss, and relay placement, thereby enabling analytical characterization of edge-node end-to-end coverage probability and its enhancement through relay placement optimization.

The main contributions of this study are summarized as follows:

(1) A coverage probability model that accounts for random traffic activation is developed for two-hop LoRa networks. Unlike methods that assume either a fixed number of active nodes or a fixed activation probability, the proposed model establishes a mapping between traffic activation intensity and the active interferer set and incorporates this mapping into the edge-terminal–relay–gateway two-hop transmission process. Path loss, thermal noise, Rayleigh fading, intra-SF interference, and residual inter-SF interference caused by imperfect SF orthogonality are jointly considered to characterize the impact of traffic-intensity variations on the interference state and coverage probability.
(2) A random-traffic-activation-driven relay placement optimization method is proposed. Existing studies primarily focus on coverage probability analysis for predefined relay locations or best-relay selection from a candidate relay set, whereas this study further investigates the impact of relay placement on coverage performance under random traffic activation. With the two-hop end-to-end coverage probability as the optimization objective, fixed, random, and optimized relay placement schemes are compared under unified system parameters, interference model, and traffic activation conditions. An iterative search is then performed to determine the relay location that yields improved coverage performance.
(3) An end-to-end reliability decomposition method is developed for decode-and-forward (DF) two-hop links. Considering the sequential transmission nature of DF relaying, the end-to-end transmission success event is decomposed into a successful first-hop decoding event and a conditional successful second-hop forwarding event. End-to-end reliability is then analyzed in terms of the first-hop coverage probability, the conditional second-hop coverage probability, and their joint impact. This decomposition helps identify link bottlenecks in two-hop LoRa networks and clarify how these bottlenecks affect edge-node coverage enhancement.
(4) Multi-scenario numerical simulations are conducted to validate the proposed model and optimization method. System performance is analyzed with respect to traffic activation intensity, relay placement strategy, optimization convergence, path-loss exponent, SINR threshold, and node density. Direct-link transmission is used as the baseline to demonstrate the effectiveness of relay-assisted transmission in enhancing edge-node coverage probability.

## 2. System Modeling and Analysis

### 2.1. Modeling and Analysis

**Figure 1:**
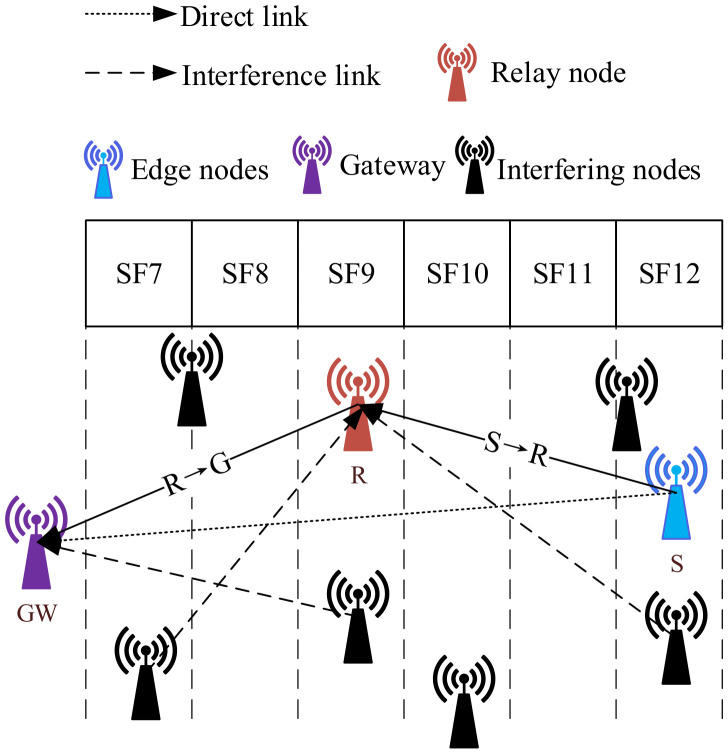
System communication model.

A simulation model for two-hop LoRa uplink communication under random traffic activation conditions is implemented in MATLAB R2024a. The considered system consists of one gateway G, one target edge source node S, one deployable relay node R, and N potential interfering terminal nodes. The gateway is deployed at the center of the network coverage area, whereas edge source nodes are placed near the coverage boundary to represent end devices with degraded link quality in long-range LoRa communication scenarios. The relay node is deployed between the edge source node and the gateway, and its placement can be optimized using the end-to-end coverage probability as the optimization objective to improve edge-node end-to-end transmission reliability. The schematic diagram of the system communication model is shown in Figure 1.

The communication architecture considered in this study includes two transmission modes: direct edge-node-to-gateway transmission and relay-assisted two-hop transmission. The SINR model jointly accounts for path loss, thermal noise, Rayleigh fading, intra-SF interference, and residual inter-SF interference arising from imperfect orthogonality among spreading factors. The two-hop transmission mode employs a DF relaying mechanism, in which packet delivery is performed in two sequential phases. In the first phase, the source node transmits its packet to the relay, and the relay must successfully decode the received packet. In the second phase, the relay forwards the decoded packet to the gateway. Accordingly, relay-to-gateway forwarding is enabled only when the first hop has been successfully completed.

The LoRa physical-layer parameters are configured in terms of the spreading factor, bandwidth, coding rate, noise figure, carrier frequency, transmit power, and receiver sensitivity threshold. A multi-SF configuration ranging from SF7 to SF12 is employed, where each SF is associated with a specific data rate, receiver sensitivity, and transmit power. In the default configuration, edge source nodes are assigned higher SFs, whereas potential interfering nodes are allocated to SF-specific regions according to their spatial locations. This configuration forms a LoRa uplink access environment in which multiple SFs coexist.

In the channel model, large-scale fading is characterized by distance-dependent path loss, whereas small-scale fading is modeled using a Rayleigh fading model. Path loss and the resulting received power are evaluated separately for the source-to-relay, relay-to-gateway, and source-to-gateway links. For interfering transmissions, the received interference power at the intended receiver is evaluated based on the distance from each interfering node to that receiver, which is either the relay or the gateway.

The interference model accounts for both intra-SF interference and residual inter-SF interference arising from imperfect orthogonality among spreading factors. For interfering nodes that use the same SF as the target signal, their received interference contributions are included in the decoding-success criterion to characterize intra-SF contention-induced interference. For interfering nodes that use SFs different from that of the target signal, their interference contributions are incorporated into the decoding-success criterion as residual inter-SF interference and are weighted according to SF-dependent decoding thresholds.

In the random traffic-activation model, a traffic-activation intensity coefficient is introduced to characterize the probability that potential interfering nodes become active under different network load levels. For the k-th SF region, the node activation probability is jointly determined by the baseline traffic-activation probability and the traffic-activation intensity coefficient, with its value constrained within a valid probability range. This formulation enables traffic-load variations to be directly reflected in the calculations of intra-SF interference, residual inter-SF interference, and end-to-end coverage probability.

### 2.2. SF-Specific Region Partitioning

(1) $$\Omega =\left\{x={\left[x,y\right]}^{T}\;|\;0\leq x\leq R_{0},\;-\frac{R_{0}}{2}\leq y\leq \frac{R_{0}}{2}\right\}$$

where Ω denotes the deployment region of potential nodes in the LoRa network; $x={\left[x,y\right]}^{T}$ is the two-dimensional position vector; and $R_{0}$ denotes the distance from the gateway to the farthest edge node, thereby characterizing the spatial extent of the network along the source-to-gateway direction. The region Ω represents a LoRa sensor network scenario with an approximately layered node deployment along this direction.

The gateway and the edge source node are located at $x_{G}={\left[0,0\right]}^{T}$ and $x_{S}={\left[R_{0},0\right]}^{T}$, respectively, while the relay-node position, denoted by $x_{R}={\left[x_{R},y_{R}\right]}^{T}$, is treated as an optimization variable. The source node is placed at the network coverage boundary to represent edge sensor nodes under weak-coverage conditions. For any two nodes A and B in the communication region, $d_{A,B}={∥x_{A}-x_{B}∥}_{2}$ denotes the link propagation distance between nodes A and B, where $x_{A}$ and $x_{B}$ are their coordinate vectors, respectively, and ${\left\|.\right\|}_{2}$ denotes the Euclidean norm

(2) $$\Phi =\left\{x_{1},x_{2},\ldots ,x_{N}\right\},\;\;x_{i}\in \Omega$$

where Φ denotes the set of potential interfering-node locations within the network coverage region; N denotes the total number of potential interfering nodes; and $x_{i}$ denotes the two-dimensional position vector of the i-th potential interfering node. Φ characterizes the potential interference topology of the network, i.e., the spatial distribution of nodes within the given deployment region that may become active for uplink transmission and interfere with the target link. This definition does not imply that all these nodes are active transmitters in every observation cycle. Since LoRa sensor nodes typically transmit packets through periodic reporting, event-triggered transmission, or random access, potential interfering nodes may be either transmitting or silent across different observation cycles. Therefore, the active interfering nodes in the current observation cycle constitute a random subset of Φ. Its cardinality and composition are determined by the subsequently defined random traffic-activation variable $a_{i}^{\left(q\right)}$ and the corresponding activation probability $p_{i}$.

To represent the layered coverage scenario along the source-to-gateway direction, the strip-shaped network region is partitioned according to the horizontal coordinate x into six SF-specific regions corresponding to SF7–SF12. The k-th SF region is defined as

(3) $$\Omega_{k}=\left\{{\left[x,y\right]}^{T}\in \Omega \;|\;\frac{\left(k-1\right)R_{0}}{6}\leq x<\frac{kR_{0}}{6}\right\},\;k=1,2,\ldots ,6$$

where $\Omega_{k}$ denotes the k-th SF-specific region, with k=1,2,…,6 corresponding to SF7–SF12, respectively, and $R_{0}$ denotes the spatial extent of the network along the source-to-gateway direction.

(4) $$k_{i}=min\left[max\left(\left\lfloor \frac{6x_{i}}{R_{0}}\right\rfloor +1,1\right),6\right]$$

where $x_{i}$ denotes the horizontal coordinate of the i-th potential interfering node, and $k_{i}\in \{1,2,\ldots ,6\}$ denotes the index of the SF-specific region to which this node belongs. This expression assigns each potential interfering node to one of the six SF-specific regions from SF7 to SF12 according to its position along the source-to-gateway direction.

The spreading factor assigned to the i-th interfering node is denoted by $s_{i}$, where $s_{i}=SF\left(6+k_{i}\right)$. Thus $k_{i}=1$ corresponds to $s_{i}=SF7$, whereas $k_{i}=6$ corresponds to $s_{i}=SF12$. Since the source node is located at the far-end boundary of the network, it is assigned SF12; hence, its SF index is set to $o_{S}=6.$ This setting characterizes a weak-coverage scenario in which the edge source node is assigned the highest SF to enhance the link budget. The SF index of the relay node, denoted by $o_{R}$, is determined by the SF-specific region in which the relay is located.

### 2.3. Channel Model and Received Power

In a two-hop LoRa uplink network, the quality of each wireless link is primarily governed by large-scale path loss and small-scale fading [30]. Large-scale path loss characterizes the distance-dependent attenuation of average signal power. By contrast, small-scale fading captures instantaneous channel-gain variations caused by multipath propagation and environmental randomness, resulting in random variations in link quality even at the same propagation distance. To characterize desired and interfering links among the source node, relay node, gateway, and potential interfering nodes in a unified manner, a generic transmission link from an arbitrary node A to node B is adopted as the basic object for channel modeling.

For the link from node A to node B, the large-scale path loss is defined as

(5) $$\Omega_{A,B}=K_{0}\theta_{A,B}d_{A,B}^{\eta }$$

where $\Omega_{A,B}$ denotes the large-scale attenuation factor of the link from node A to node B; $K_{0}$ is the reference path-loss constant; $\theta_{A,B}$ is the link-scale correction factor; $d_{A,B}$ is the distance between nodes A and B; and η is the path-loss exponent. This expression maps the geometric link distance to large-scale propagation attenuation and provides a unified basis for calculating the received powers of both desired and interfering links. In the subsequent numerical simulations, the path-loss exponent η is set to 2.70 as the baseline value to represent a moderately obstructed urban or suburban LoRa propagation environment rather than an ideal free-space propagation condition.

(6) $$K_{0}={\left(\frac{4\pi f_{c}}{c}\right)}^{2}$$

where $f_{c}$ is the carrier frequency of the LoRa system, and c is the speed of light. $K_{0}$ denotes the reference path-loss constant, which characterizes the carrier-frequency-dependent free-space attenuation at the reference distance. According to the free-space propagation model, the free-space path loss at the reference distance increases with the carrier frequency. Therefore, $K_{0}$ incorporates the carrier-frequency-dependent loss component into the large-scale attenuation model. For the link from node A to node B, the large-scale attenuation factor $\Omega_{A,B}$ is determined jointly by the reference path-loss constant $K_{0}$, the link distance $d_{A,B}$, the path-loss exponent η and the link-scale correction factor $\theta_{A,B}$.

A Rayleigh small-scale fading model is adopted to characterize rapid channel fluctuations of LoRa links in multipath propagation environments. The small-scale fading power gain of the link from node A to node B, denoted by $H_{A,B}$ is assumed to follow an exponential distribution with unit mean, i.e., $H_{A,B}∼Exp(1)$. The Rayleigh fading assumption improves analytical tractability by enabling a unified integration of the channel model with Bernoulli random traffic activation, weighted intra-/inter-SF interference, and the Laplace transform of aggregate interference. This formulation provides an analytical foundation for deriving the single-hop coverage probability and the DF-based two-hop joint coverage probability. Accordingly, Rayleigh fading is adopted as the baseline channel model to investigate the effects of traffic-activation intensity, SF interference structure, and relay position on the coverage reliability of edge LoRa nodes.

(7) $$P_{r,A,B}=\frac{P_{A}H_{A,B}}{\Omega_{A,B}}$$

where $P_{r,A,B}$ denotes the instantaneous received power at node B from the signal transmitted by node A, and $P_{A}$ denotes the transmit power of node A. This expression indicates that the instantaneous received power of a link is jointly determined by the transmit power, large-scale attenuation, and small-scale fading. An increase in $\Omega_{A,B}$ indicates more severe propagation attenuation over the link, resulting in a lower received signal power. Conversely, a larger $H_{A,B}$ corresponds to more favorable instantaneous channel conditions and therefore leads to a higher received power. Thus, this expression provides a unified received-power model for the subsequent coverage-probability analysis of the source-to-relay, relay-to-gateway, and direct source-to-gateway links.

The receiver thermal noise power expressed in dBm is given by

(8) $$\sigma_{dBm}^{2}=-174+NF+10{log}_{10}\left(BW\right)$$

where −174 dBm/Hz denotes the assumed thermal noise power spectral density at room temperature, NF denotes the receiver noise figure, and BW denotes the system bandwidth expressed in Hz.

During the first transmission phase, the source node S transmits a signal to the relay node R. The signal received at the relay node R is given by

(9) $$y_{R}=\sqrt{P_{S}}h_{S,R}x_{S}+\sum_{i=1}^{N}a_{i}^{\left(q\right)}\sqrt{P_{ki}}h_{i,R}x_{i,R}+n_{R}$$

where $P_{S}$ denotes the transmit power of the source node; $h_{S,R}$ denotes the complex channel coefficient from the source node to the relay; $x_{S}$ denotes the signal transmitted by the source node; $P_{ki}$ denotes the transmit power associated with the SF region of the i-th potential interfering node; $h_{i,R}$ denotes the complex channel coefficient from this interfering node to the relay; $x_{i,R}$ denotes the unit-power signal transmitted by this node in the first phase; $n_{R}$ denotes the additive white gaussian noise at the relay; and $a_{i}^{\left(q\right)}$ denotes the activation state of the i-th potential interfering node during the q-th observation cycle. In the second phase, if the message from the source node is successfully decoded at relay R, the decoded message is remodulated and forwarded to gateway G. The received signal at the gateway is expressed as

(10) $$y_{G}=\sqrt{P_{R}}h_{R,G}x_{R}+\sum_{i=1}^{N}a_{i}^{\left(q\right)}\sqrt{P_{ki}}h_{i,G}x_{i,G}+n_{G}$$

where $P_{R}$ denotes the transmit power of the relay; $h_{R,G}$ denotes the complex channel coefficient from the relay to the gateway; $x_{R}$ denotes the signal forwarded by the relay; $h_{i,G}$ denotes the complex channel coefficient from the i-th interfering node to the gateway; $x_{i,G}$ denotes the unit-power signal transmitted by this node in the second phase; and $n_{G}$ denotes the additive white gaussian noise at the gateway.

### 2.4. SNR Demodulation Thresholds and SF Rejection Thresholds

In a LoRa uplink sensor network, nodes typically transmit packets through periodic reporting, event-triggered transmission, or random access. Therefore, potential interfering nodes may be active or silent in different observation cycles. To characterize how traffic-load variations affect the interference field, the system operation time is divided into consecutive discrete observation cycles. Here, q denotes the observation-cycle index, and $T_{in}$ denotes the duration of each observation cycle. During the q-th observation cycle, each potential interfering node may be either transmitting or silent. Therefore, a Bernoulli random traffic-activation variable is introduced to characterize the traffic-activation state of each node at the observation-cycle level.

(11) $$a_{i}^{\left(q\right)}=\left\{\begin{array}{ll} 1, & Node\;i\;is\;active\;for\;transmission\;during\;observation\;cycle\;q\\ 0, & Node\;i\;remains\;silent\;during\;observation\;cycle\;q \end{array}\right.\;$$

where $a_{i}^{\left(q\right)}$ denotes the traffic activity indicator of node i during observation cycle q. This indicator is used to distinguish potentially interfering nodes from those that are active for transmission and therefore contribute to the aggregate interference.

For the q-th observation cycle, the traffic activity of the i-th potential interferer is represented by the Bernoulli random variable $a_{i}^{\left(q\right)}$, where $a_{i}^{\left(q\right)}∼Bernoulli(p_{i})$, $p_{i}$ denotes the probability that node i is active for transmission during an observation cycle. Specifically, $a_{i}^{\left(q\right)}=1$ indicates that node i is active for transmission during observation cycle q and may interfere with the target link, whereas $a_{i}^{\left(q\right)}=0$ indicates that node i remains silent and does not contribute to the aggregate interference in that period. The proposed cycle-level stochastic traffic activity model enables the intermittent uplink traffic behavior of LoRa nodes to be explicitly incorporated into interference-field modeling, thereby distinguishing potential interferers from actually active interferers.

Since different SF regions are associated with different data rates, time-on-air durations, and channel occupancy levels, the cycle-level activation probability of each node is modeled as an SF-dependent parameter. Specifically, if the i-th potential interferer belongs to SF region $k_{i}$, its activation probability is denoted by $p_{i}=p_{k_{i}}(\lambda_{A})$. Where $\lambda_{A}$ denotes the normalized traffic activation intensity, which characterizes the overall traffic load, and $p_{k_{i}}(\lambda_{A})$ represents the activation probability of nodes in SF region $k_{i}$ under the corresponding traffic load condition. Accordingly, different activation probabilities are assigned to nodes in different SF regions based on their transmission rates and channel occupancy characteristics. This treatment enables the stochastic traffic activation model to capture the spatial heterogeneity of traffic loads in multi-SF LoRa networks.

(12) $$p_{k}\left(\lambda_{A}\right)=min\left(\lambda_{A}p_{A}^{k},1\right)$$

where $p_{A}^{k}$ denotes the baseline activation probability of SF region k and $\lambda_{A}$ represents the traffic-load scaling factor. This mapping determines the resulting activation probability from the traffic-load-scaled baseline probability, with min(⋅,1) constraining the probability to be no greater than one.

To characterize traffic-occupancy heterogeneity across SF regions, the baseline traffic activation probability for SF region k is defined as $p_{A}^{k}=min\left(L_{pac}/(R_{b,k}T_{in}),1\right)$. Where $L_{pac}$ denotes the length of a single uplink packet, $R_{b,k}$ denotes the data rate of SF region k, and $T_{in}$ denotes the duration of the observation cycle. This probability characterizes the equivalent channel-occupancy ratio of a node within an observation cycle. A shorter time-on-air leads to a lower activation probability, whereas the probability is capped at unity when the time-on-air approaches or exceeds the observation cycle. Since higher SFs generally result in lower data rates and longer time-on-air values, the corresponding baseline traffic activation probabilities are typically higher. This formulation therefore captures traffic-occupancy heterogeneity across SF regions.

(13) $$R_{b,k}=\frac{{SF}_{k}\cdot CR\cdot BW}{2^{{SF}_{k}}}$$

where $SF_{k}$, CR and BW denote the spreading factor assigned to SF region k, the coding rate and the system bandwidth, respectively. This expression indicates that a higher spreading factor reduces the data rate, thereby increasing the time-on-air and the baseline traffic activation probability.

(14) $$\Phi_{a}^{\left(q\right)}=\left\{x_{i}\in \Phi \;|\;a_{i}^{\left(q\right)}=1\right\}$$

where $\Phi_{a}^{\left(q\right)}$ denotes the set of active interferers during observation cycle q. This set consists of the nodes in the potential interferer set Φ whose activation indicator satisfies $a_{i}^{\left(q\right)}=1$ and therefore constitutes a random subset of Φ. The cardinality and composition of $\Phi_{a}^{\left(q\right)}$ vary with the traffic activation states, thereby reflecting the cycle-level randomness of the interference field in the LoRa uplink network. This set couples the spatial topology of nodes with the stochastic traffic activation process, thereby providing a basis for the subsequent analysis of co-SF interference, inter-SF residual interference, and coverage probability.

(15) $$E\left[N_{a,k}^{\left(q\right)}\right]=N_{k}p_{k}\left(\lambda_{A}\right)$$

where $N_{a,k}^{\left(q\right)}$ denotes the number of active interferers in SF region k during observation cycle q and $N_{k}$ denotes the number of potential interferers in that region. This expression indicates that a higher traffic activation intensity results in a larger expected number of active interferers.

The above stochastic traffic activation model establishes a direct link between traffic-load variations, the LoRa interference field, and coverage probability. Specifically, the traffic activation intensity $\lambda_{A}$ determines the SF-dependent activation probability $p_{k}(\lambda_{A})$, which in turn governs the active interferer set $\Phi_{a}^{\left(q\right)}$ during observation cycle q. This active set further determines the aggregate same-SF interference $I_{same}$ and the inter-SF residual interference $I_{cross}$, thereby affecting the coverage probability $P_{cov}$ of the target link. Table 1 presents the symbols and definitions of key parameters.

## 3. Analytical Modeling of Same-SF Interference and Inter-SF Residual Interference

### 3.1. Determination of the Target SF and Interfering SF

In a multi-SF LoRa uplink network, the interference category depends not only on whether the interfering node is active for transmission but also on the spreading factors assigned to the target and interfering links. For an arbitrary target link A→B, $o_{A,B}$ denotes the SF index of the target link, whereas $k_{i}$ denotes the SF-region index of the i-th interfering node. The index $o_{A,B}$ denotes the SF index assigned to the target link A→B, whereas $k_{i}$ denotes the SF-region index determined by the location of the i-th interfering node. Comparing $o_{A,B}$ with $k_{i}$ allows the interference from node i to link A→B to be classified as either same-SF interference or inter-SF residual interference.

When $k_{i}=o_{A,B}$, the interfering node and the target link are assigned the same SF, and the interference from this node to link A→B is classified as same-SF interference. In contrast, when $k_{i}\neq o_{A,B}$, they are assigned different SFs, and the corresponding interference is classified as inter-SF residual interference.

In the proposed dual-hop LoRa transmission model, the target SF of each link is determined by the SF region of the corresponding transmitting node. For the first hop S→R, the desired signal originates from source node S at the network edge. Since this source node is assigned SF12, the target SF index of the first hop is given by $o_{S,R}=o_{S}=6$ according to the adopted SF-index mapping. For the direct source-to-gateway link S→G, the desired signal also originates from source node S. According to the adopted SF-index mapping, the target SF index of this link is therefore given by $o_{S,G}=o_{S}=6$. For the second hop R→G, the desired signal originates from relay node R. Accordingly, the target SF index of this hop is determined by the SF region of R and is denoted by $o_{R,G}=o_{R}$.

In this study, a LoRa SF rejection-threshold matrix is employed to provide a unified representation of same-SF interference and inter-SF residual interference. In this matrix, each row corresponds to a target SF, whereas each column corresponds to an interfering SF. Table 2 cites Equation (6) in Reference [27].

Table 2 presents the SF rejection-threshold matrix. Its dB-domain element is denoted by $\Delta_{dB}(o,k)$, where o and k denote the SF indices of the target link and the interfering node, respectively. The dB-domain rejection threshold $\Delta_{dB}(o,k)$ can be converted into the corresponding linear-domain interference weight as follows:

(16) $$\delta_{o,k}=10^{\frac{\Delta_{dB}\left(o,k\right)}{10}}$$

where $\delta_{o,k}$ denotes the linear-domain equivalent interference weight corresponding to target SF index o and interfering SF index k. This weight is derived from the dB-domain SF rejection threshold and characterizes the non-ideal orthogonality among multi-SF LoRa signals, thereby quantifying the corresponding residual interference contribution. In Table 2, the rows correspond to the SF assigned to the target link, whereas the columns correspond to the SF assigned to the interfering node. The matrix element $\Delta_{dB}(o,k)$ denotes the equivalent rejection threshold associated with target SF index o and interfering SF index k. The diagonal elements of the matrix characterize same-SF interference, whereas the off-diagonal elements characterize inter-SF residual interference. By mapping each dB-domain element to the corresponding linear-domain weight $\delta_{o,k}$, the interference contributions associated with different SF combinations can be uniformly incorporated into the expressions for aggregate interference and coverage probability. This formulation captures the impact of non-ideal orthogonality among LoRa SFs on coverage performance.

### 3.2. Aggregate Modeling of Same-SF Interference and Inter-SF Residual Interference

For an arbitrary target link A→B, receiver B experiences aggregate interference from active interferers during observation cycle q while demodulating the desired signal. The aggregate interference is jointly determined by node traffic activation states, transmit powers, path losses of the interfering links, small-scale fading gains, and SF-dependent rejection weights between the target and interfering SFs. Accordingly, these factors are jointly incorporated into the aggregate interference model to characterize the impact of stochastic traffic activation and non-ideal multi-SF orthogonality on the coverage performance of the target link. For the target link A→B, the aggregate interference power observed at receiver B can be written as

(17) $$I_{B}\left(A\right)=\sum_{i=1}^{N}a_{i}^{\left(q\right)}\frac{P_{k_{i}}H_{i,B}\delta_{o_{A,B},k_{i}}}{\Omega_{i,B}}$$

where $I_{B}(A)$ denotes the aggregate interference power observed at receiver B for the target link A→B, $P_{k_{i}}$ denotes the transmit power of the interfering node associated with SF region $k_{i}$, $H_{i,B}$ denotes the Rayleigh fading gain between interfering node i and receiver B, and $\Omega_{i,B}$ denotes the large-scale attenuation factor of the corresponding interfering link.

Accordingly, the aggregate interference at receiver B is decomposed into same-SF interference and inter-SF residual interference, i.e., $I_{B}(A)=I_{B,same}(A)+I_{B,cross}(A)$. Where $I_{B,same}\left(A\right)$ denotes the interference contributed by active nodes assigned the same SF as the target link A→B, whereas $I_{B,cross}\left(A\right)$ denotes the residual interference contributed by active nodes assigned different SFs from the target link due to non-ideal orthogonality among SFs. This decomposition enables the distinction among three interference modes: same-SF interference only, inter-SF residual interference only, and the coexistence of both interference components.

(18) $$I_{B,same}\left(A\right)=\sum_{\begin{array}{c} i=1\\ k_{i}=o_{A,B} \end{array}}^{N}a_{i}^{\left(q\right)}\frac{P_{k_{i}}H_{i,B}\delta_{o_{A,B},k_{i}}}{\Omega_{i,B}}$$

where the same-SF interference is contributed only by nodes satisfying $k_{i}=o_{A,B}$, namely, interfering nodes assigned the same SF as the target link.

(19) $$I_{B,cross}\left(A\right)=\sum_{\begin{array}{c} i=1\\ k_{i}\neq o_{A,B} \end{array}}^{N}a_{i}^{\left(q\right)}\frac{P_{k_{i}}H_{i,B}\delta_{o_{A,B},k_{i}}}{\Omega_{i,B}}$$

where the inter-SF residual interference is contributed by all active nodes satisfying $k_{i}\neq o_{A,B}$. This formulation shows that inter-SF interference is not neglected in the proposed model; rather, it is incorporated as residual interference through the off-diagonal elements of the rejection-threshold matrix.

## 4. Single-Hop Coverage Probability Analysis

### 4.1. Reception Threshold and Noise Term

For the target link A→B, the coverage performance depends not only on the desired signal power and aggregate interference but also on the reception threshold corresponding to the SF assigned to the target link. Different SFs are associated with different data rates and receiver sensitivities. In general, higher SFs correspond to lower demodulation thresholds but require longer time-on-air durations. The SF index assigned to the target link A→B is denoted by $o_{A,B}$, whereas the corresponding linear-domain reception threshold is denoted by $q_{o_{A,B}}$. This threshold specifies the minimum reception condition required for the target link to be successfully demodulated in the presence of aggregate interference and thermal noise, and its value is determined from the SF-dependent threshold table:

(20) $$q_{o}=10^{\frac{Q_{dB}\left(o\right)}{10}}$$

where $Q_{dB}\left(o\right)$ denotes the dB-domain reception threshold associated with SF index o, whereas $q_{o}$ denotes the corresponding linear-domain threshold. This threshold specifies the minimum reception condition required for successful demodulation under different SF configurations.

The SF reception threshold characterizes the minimum demodulation requirement for the target link under thermal noise, whereas the distinction between same-SF interference and inter-SF residual interference is captured by the weighting factors derived from the SF rejection-threshold matrix. The threshold $q_{o_{A,B}}$ is therefore introduced in the coverage-probability derivation to construct the normalized noise term for link A→B and map the thermal-noise power onto the channel-gain decision scale of the target link. The normalized noise term for link A→B is defined as

(21) $$b_{A,B}=\frac{q_{o_{A,B}}\Omega_{A,B}}{P_{A}}\sigma^{2}$$

where $b_{A,B}$ denotes the normalized noise term for link A→B, $\sigma^{2}$ denotes the receiver thermal-noise power, and $P_{A}$ denotes the transmit power of node A. This expression maps the thermal-noise power onto the same decision scale as the Rayleigh channel gain.

### 4.2. Analysis of Equivalent Interference Weights and Single-Hop Coverage Probability

The equivalent interference coefficient of the i-th interfering node with respect to target link A→B is defined as follows:

(22) $$\alpha_{A,B,i}=\delta_{o_{A,B},k_{i}}\frac{P_{k_{i}}\Omega_{A,B}}{P_{A}\Omega_{i,B}}$$

where $\alpha_{A,B,i}$ denotes the normalized interference strength of the i-th interfering node with respect to target link A→B. $\delta_{o_{A,B},k_{i}}$ denotes the SF-dependent rejection weight between the target SF and the interfering SF. $P_{A}$ denotes the transmit power of the target transmitter. $\Omega_{A,B}$ and $\Omega_{i,B}$ denote the large-scale attenuation factors of the target link and the interfering link, respectively. A larger value of $\alpha_{A,B,i}$ indicates a stronger degradation effect of the corresponding interfering node on the coverage probability of the target link.

(23) $${\tilde{\alpha }}_{A,B,i}=\left\{\begin{array}{ll} \alpha_{A,B,i}, & the\;coexistence\;of\;same-SF\;and\;inter-SF\;interference\\ \alpha_{A,B,i}1\left(k_{i}=o_{A,B}\right), & same-SF\;interference\;only\\ \alpha_{A,B,i}1\left(k_{i}\neq o_{A,B}\right), & inter-SF\;interference\;only \end{array}\right.$$

where ${\tilde{\alpha }}_{A,B,i}$ denotes the equivalent interference coefficient after interference-mode selection. 1(⋅) is an indicator function that determines whether a potential interfering node is retained in the interference calculation under the corresponding interference mode. This expression supports three simulation settings within a unified formulation: the complete-system setting, the same-SF-only benchmark and the inter-SF-only benchmark. It therefore provides a mathematical basis for comparing the effects of different interference modes on the single-hop coverage probability.

The coverage-probability factor associated with the i-th potential interferer under Bernoulli random traffic activation and Rayleigh fading of the interfering channel is given as follows:

(24) $$F_{A,B,i}=1-p_{i}+\frac{p_{i}}{1+{\tilde{\alpha }}_{A,B,i}}$$

where $F_{A,B,i}$ denotes the Laplace-transform factor associated with the contribution of the i-th potential interferer to the coverage probability of link A→B. The first term ($1-p_{i}$) corresponds to the inactive state of the node, in which no interference is generated. The second term captures the average contribution of the activated node under Rayleigh fading of the interfering channel. The detailed derivation of Equation (24) is provided in Appendix B.

The average coverage probability of link A→B is given by

(25) $$P_{A,B}=exp\left(-b_{A,B}\right)\prod_{i=1}^{N}\left[1-p_{i}+\frac{p_{i}}{1+{\tilde{\alpha }}_{A,B,i}}\right]$$

where $P_{A,B}$ denotes the average coverage probability of link A→B. The exponential term captures the effect of thermal noise on the successful transmission probability of the link. The product term characterizes the joint impact of all potential interfering nodes under random traffic activation and SF-dependent interference weighting.

To further characterize the stratified impact of potential interfering nodes in different SF regions on the coverage performance of the target link, the product term in Equation (25) is reorganized according to the SF region to which each node belongs. Equation (25) can therefore be equivalently rewritten in an SF-grouped form as follows:

(26) $$P_{A,B}=exp\left(-b_{A,B}\right)\prod_{k=1}^{6}\prod_{x_{i}\in \Omega_{k}}\left[1-p_{k}\left(\lambda_{A}\right)+\frac{p_{k}\left(\lambda_{A}\right)}{1+{\tilde{\alpha }}_{A,B,i}}\right]$$

where the inner product term represents the joint impact of the potential interfering nodes in the k-th SF region on the coverage probability of link A→B. The outer product term aggregates the SF-region-level interference contributions across the six SF regions spanning SF7 to SF12. The interference terms in Equation (26) are reorganized according to SF-region partitioning to explicitly characterize the coupling among random traffic activation, SF-region partitioning, same-SF interference, and SF-weighted residual inter-SF interference. This formulation therefore provides a clearer representation of how the SF-dependent interference structure affects the single-hop coverage probability in a multi-SF LoRa network with random activation. The detailed derivations of Equations (25) and (26) are provided in Appendix A.

## 5. DF-Based Dual-Hop Coverage Probability Analysis

### 5.1. Dual-Hop Joint Coverage Probability

A DF relay mechanism is adopted to establish a dual-hop LoRa transmission link. The source node S transmits a packet to the relay node R in the first transmission phase. If R successfully decodes the packet from S, the decoded packet is forwarded to the gateway G in the second transmission phase. Since the first hop S→R and the second hop R→G are transmitted over separate channels and their desired signals are separated in the time domain, no mutual interference is generated at the receivers.

The event in which the relay node R successfully decodes the packet transmitted by the source node S is denoted by $C_{S,R}$. This event indicates that the first-hop link S→R satisfies the successful-reception condition and serves as the prerequisite for subsequent second-hop forwarding. The event in which the relay node R successfully decodes the data from the source node S is denoted by $C_{R,G}$.

End-to-end transmission is successful only when both $C_{S,R}$ and $C_{R,G}$ occur. The successful dual-hop transmission event can therefore be expressed as $D=C_{S,R}\cap C_{R,G}$.

The normalized noise terms corresponding to the first-hop and second-hop links are given as follows:

(27) $$b_{S,R}=\frac{q_{o_{S,R}}\Omega_{S,R}}{P_{S}}\sigma^{2}\;,\;b_{R,G}=\frac{q_{o_{R,G}}\Omega_{R,G}}{P_{R}}\sigma^{2}$$

where $P_{S}$ denotes the transmit power of the source node S, and $P_{R}$ denotes the transmit power of the relay node R. The corresponding target SF indices are $o_{S,R}=6$ and $o_{R,G}=o_{R}$. These two normalized noise terms quantify the minimum reception requirements imposed by thermal noise on the first-hop and second-hop links.

The transmitting node, receiving node, and target SF may differ between the first-hop and second-hop links. Consequently, the same potential interfering node may have different equivalent interference contributions to the two links. The equivalent interference coefficients of the i-th interfering node with respect to the first hop S→R and the second hop R→G are defined as follows:

(28) $$\alpha_{S,R,i}=\delta_{o_{S,R},k_{i}}\frac{P_{k_{i}}\Omega_{S,R}}{P_{S}\Omega_{i,R}}\;,\;\alpha_{R,G,i}=\delta_{o_{R,G},k_{i}}\frac{P_{k_{i}}\Omega_{R,G}}{P_{R}\Omega_{i,G}}$$

where $\alpha_{S,R,i}$ denotes the normalized interference strength of the i-th interfering node with respect to the first hop S→R. Similarly, $\alpha_{R,G,i}$ denotes the normalized interference strength of the same interfering node with respect to the second hop R→G.

The traffic activation states of the potential interfering nodes are assumed to remain unchanged across the two transmission phases within a given traffic observation cycle. The end-to-end coverage probability of DF dual-hop transmission can then be expressed as follows:

(29) $$P_{D}=exp\left(-b_{S,R}-b_{R,G}\right)\prod_{i=1}^{N}\left[1-p_{i}+\frac{p_{i}}{\left(1+{\tilde{\alpha }}_{S,R,i}\right)\left(1+{\tilde{\alpha }}_{R,G,i}\right)}\right]$$

where $P_{D}$ denotes the end-to-end coverage probability of DF dual-hop transmission. It corresponds to the joint probability that the first-hop transmission over S→R and the second-hop transmission over R→G are both successful. The detailed derivation of Equation (29) is provided in Appendix C.

Because the activation state of the i-th potential interfering node affects both hops within the same traffic-state cycle, the same $p_{i}$ in the product term is used to characterize the random activation effect of this node on the joint dual-hop event. The second-hop conditional forwarding reliability is introduced to identify the performance bottleneck of the dual-hop link and is defined as $P_{R,G|S,R}=P_{D}/P_{S,R}$. This metric represents the probability of successful second-hop forwarding conditioned on successful first-hop decoding. Therefore, the dual-hop coverage probability can be further expressed as $P_{D}=P_{S,R}P_{R,G|S,R}$. $P_{S,R}$ can be obtained from Equation (23).

### 5.2. Direct-Link and Dual-Hop Cooperative Analysis

The direct-link coverage event from the source node S to the gateway G is introduced to characterize the complementary roles of direct transmission and relay forwarding in terms of coverage performance. This event is defined as $C_{S,G}=\left\{S\to G\;\mathrm{succeeds}\right\}$. The event $C_{S,G}$ denotes successful reception and decoding of the uplink packet from S at G without assistance from the relay node R. The source node is configured with SF12 and the corresponding SF index is given by $o_{S,G}=6$. When both direct transmission and DF dual-hop relaying are considered, the cooperative coverage event of the system can be expressed as $C_{coop}=C_{S,G}\cup D$. The event $C_{coop}$ denotes the coverage event in which at least one of the direct link and the DF dual-hop relay link is successful. Since coverage can be achieved through either the direct source-to-gateway link or the DF relaying path, the cooperative coverage probability of the system is defined as the union probability of the direct-coverage event and the dual-hop coverage event. It is given by

(30) $$P_{C}=P_{S,G}+P_{D}-P_{J}$$

where $P_{C}$ denotes the cooperative coverage probability, $P_{S,G}$ denotes the direct-coverage probability, $P_{D}$ denotes the dual-hop coverage probability, and $P_{J}$ denotes the joint probability that the direct-coverage event and the dual-hop coverage event are simultaneously successful.

(31) $$P_{J}=P\left(C_{S,G}\cap C_{S,R}\cap C_{R,G}\right)$$

where $P_{J}$ denotes the joint probability that the direct-coverage event and the dual-hop coverage event occur simultaneously and is subtracted to avoid double counting.

Under the assumptions of Bernoulli traffic activation and Rayleigh fading, the joint coverage probability over the three links is given by

(32) $$P_{J}=exp\left(-b_{S,G}-b_{S,R}-b_{R,G}\right)\prod_{i=1}^{N}\left[1-p_{i}+\frac{p_{i}}{\left(1+{\tilde{\alpha }}_{S,G,i}\right)\left(1+{\tilde{\alpha }}_{S,R,i}\right)\left(1+{\tilde{\alpha }}_{R,G,i}\right)}\right]$$

where ${\tilde{\alpha }}_{S,G,i},$ ${\tilde{\alpha }}_{S,R,i}$ and ${\tilde{\alpha }}_{R,G,i}$ denote the equivalent interference coefficients associated with interfering node i on the direct S→G. link, the first-hop S→R. link, and the second-hop R→G. link, respectively. This expression indicates that the direct-link and dual-hop coverage events are statistically coupled because they are governed by the same potential interferer topology and random traffic activation states. The detailed derivations of Equations (30)–(32) are provided in Appendix D.

## 6. Random-Traffic-Aware Relay Placement Optimization

### 6.1. Optimization Objective

The relay position $x_{R}$ is a critical design variable in the optimization of the dual-hop coverage probability. Variations in $x_{R}$ directly affect the source-to-relay distance $d_{S,R}$, the relay-to-gateway distance $d_{R,G}$, and the distance $d_{i,R}$ from the i-th interfering node to the relay. In addition, $x_{R}$ determines the SF region assigned to the relay and the target SF adopted for the second hop.

To ensure physically feasible relay placement, constraints are imposed on the feasible deployment region of the relay node. The feasible relay deployment region is defined as $\Omega_{R}=\left\{x_{R}\in \Omega \;|\;d_{S,R}\geq d_{min},\;d_{R,G}\geq d_{min}\right\}$ denotes the feasible region that satisfies the relay deployment constraints and $d_{min}$ denotes the minimum separation distance. This constraint requires the relay node to maintain a minimum distance of $d_{min}$ from both the source node and the gateway. It prevents unrealistically short links during numerical optimization and preserves the physical validity of the path-loss calculation and the coverage probability model.

(33) $$T=\left\{T_{1},T_{2},\ldots ,T_{M}\right\}$$

where T denotes the set of random topology realizations used for relay optimization; M denotes the number of training topologies; and $T_{M}$ denotes the M-th topology realization of the potential interfering nodes.

To improve the robustness of the relay placement optimization results in random network environments, the relay position is not optimized for a single fixed topology. Instead, each candidate relay position is evaluated by averaging the dual-hop coverage probability over multiple random topology realizations. The objective function is defined as

(34) $$J\left(x_{R}\right)=\frac{1}{M}\sum_{m=1}^{M}P_{D}\left(x_{R};T_{m},\lambda_{A}\right)$$

where $J\left(x_{R}\right)$ denotes the average end-to-end dual-hop coverage probability obtained for relay position $x_{R}$ over a set of random topology realizations and $\lambda_{A}$ denotes the traffic activation intensity. Averaging the dual-hop coverage probability over different topology realizations mitigates the dependence of the optimization result on any specific topology and improves the statistical robustness of relay deployment under random traffic activation and random node distributions.

To determine the relay deployment position that provides the maximum coverage gain, the average dual-hop coverage performance function $J\left(x_{R}\right)$ is maximized over the feasible relay deployment region $\Omega_{R}$. The optimization problem is formulated as

(35) $$x_{R}^{⋆}=arg\underset{x_{R}\in \Omega_{R}}{max}J\left(x_{R}\right)$$

where $x_{R}^{⋆}$ denotes the optimal relay position that maximizes the average dual-hop coverage probability over the random topology set.

### 6.2. Partition-Based Projected Gradient Search

To incorporate the SF partitioning characteristics of LoRa networks, the feasible relay deployment region $\Omega_{R}$ is divided into six candidate subregions corresponding to SF7–SF12. This partitioning enables the relay placement optimization to jointly account for the deployment position, the target SF selection, and the SF-weighted interference structure when evaluating the dual-hop coverage probability.

(36) $$\Omega_{R}=\underset{l=1}{\overset{6}{⋃}}\Omega_{R,l}$$

where $\Omega_{R}$ denotes the feasible set of relay positions within the l-th SF region. In the implementation, an intra-partition margin is introduced to prevent the relay from being placed on partition boundaries or in close proximity to the source node or the gateway.

To further incorporate the SF partitioning constraints into relay placement optimization, a partition-wise optimal relay position is identified within each of the six candidate SF partitions. For the l-th SF partition, the corresponding candidate optimal relay position is defined as

(37) $$x_{R,l}^{⋆}=arg\underset{x_{R}\in \Omega_{R,l}}{max}J_{l}\left(x_{R}\right)$$

where $\Omega_{R,l}$ denotes the feasible relay deployment region within the l-th candidate SF partition, and $J_{l}\left(x_{R}\right)$ denotes the average dual-hop coverage probability associated with relay position $x_{R}$ in this partition. This partition-wise optimization approach enables the relay deployment performance to be evaluated separately for different SF partitions while preserving the SF-region constraints. Finally, the globally optimal relay deployment solution is obtained by selecting the candidate position with the highest average dual-hop coverage probability among the six partition-wise optima.

The numerical gradient of the objective function is estimated using a central finite-difference method. For a given relay position $x_{R}=[x_{R},y_{R}{]}^{T}$, the partial derivatives of $J_{l}\left(x_{R}\right)$ with respect to the x- and y-coordinates can be approximated as follows:

(38) $$\frac{\partial J_{l}}{\partial x_{R}}\approx \frac{J_{l}(x_{R}+\varrho_{g},y_{R})-J_{l}(x_{R}-\varrho_{g},y_{R})}{2\epsilon_{g}}$$

(39) $$\frac{\partial J_{l}}{\partial y_{R}}\approx \frac{J_{l}(x_{R},y_{R}+\varrho_{g})-J_{l}(x_{R},y_{R}-\varrho_{g})}{2\varrho_{g}}$$

where $\varrho_{g}$ denotes the finite-difference perturbation step size. By applying small positive and negative perturbations along the two coordinate directions, this method estimates the local variation trend of the objective function at the current relay position. The resulting numerical gradient direction is then used to update the relay position.

At the t-th iteration, a normalized gradient ascent strategy is adopted to update the relay position toward a region with improved coverage performance. Specifically, the candidate relay position is obtained by moving from the current point $x_{R}^{\left(t\right)}$ along the ascent direction of the objective function $J_{l}\left(x_{R}\right)$, i.e.,

(40) $${\tilde{x}}_{R}^{\left(t+1\right)}=x_{R}^{\left(t\right)}+\mu_{t}\frac{\nabla J_{l}\left(x_{R}^{\left(t\right)}\right)}{{∥\nabla J_{l}\left(x_{R}^{\left(t\right)}\right)∥}_{2}}$$

where $x_{R}^{\left(t\right)}$ denotes the relay position at the t-th iteration; $\mu_{t}$ denotes the current search step size; and $\nabla J_{l}\left(x_{R}^{\left(t\right)}\right)$ denotes the numerical gradient of the objective function evaluated at $x_{R}^{\left(t\right)}$. This update moves the relay position in the direction of increasing coverage probability.

(41) $$x_{R}^{\left(t+1\right)}=\Pi_{\Omega_{R,l}}\left({\tilde{x}}_{R}^{\left(t+1\right)}\right)$$

where $\Pi_{\Omega_{R,l}}\left(\cdot \right)$ denotes the projection operator onto the l-th feasible relay deployment partition. This projection ensures that the updated relay position remains within the feasible partition while satisfying the boundary and minimum-distance constraints.

To improve the stability of the iterative relay placement optimization process, a backtracking line search mechanism is introduced when the candidate position does not increase the objective function value. If the candidate relay position obtained from (40) does not increase the value of $J_{l}\left(x_{R}\right)$, the current search step size is reduced as $\zeta \mu_{t}\to \mu_{t}$, where 0<ζ<1, and the candidate position is regenerated. This backtracking step prevents an excessively large step size from driving the search away from a promising ascent direction. The algorithm is terminated when the absolute change in the objective function between two consecutive iterations satisfies $\left|J_{l}\left(x_{R}^{\left(t+1\right)}\right)-J_{l}\left(x_{R}^{\left(t\right)}\right)\right|\leq \varepsilon$, or when the maximum number of iterations $T_{max}$ is reached, where ζ denotes the step-size contraction factor, ε denotes the convergence tolerance, and $T_{max}$ denotes the maximum number of iterations. This criterion determines whether the relay placement optimization has converged or reached the preset computational limit.

Let K=6, M, N, $T_{max}$ and $n_{g}$ denote the number of candidate SF partitions, the number of training topologies, the number of potential interfering nodes in each topology, the maximum number of iterations for each partition, and the number of objective-function evaluations required for each gradient estimate, respectively. The dominant computational complexity of the relay placement optimization procedure is then given by

(42) $$O\left(K\cdot M\cdot N\cdot T_{max}{\cdot n}_{g}\right)$$

where the computational complexity is mainly determined by the numbers of SF partitions, topology realizations, potential interfering nodes, maximum iterations and objective-function evaluations required for each gradient estimate.

The proposed method may face certain limitations as the node density within the coverage area increases. As the network scale grows, especially in high-density node scenarios, the evaluation of the objective function and the gradient-based search process may introduce considerable computational overhead. Therefore, the proposed relay placement optimization method is more suitable for offline or semi-offline network planning and relay deployment optimization than for real-time execution during each LoRa packet transmission or each traffic observation cycle.

In practical LoRa networks, relay locations, terminal spatial distributions, SF-region partitioning, and long-term traffic loads usually vary relatively slowly. Accordingly, the optimization process can be performed in advance by a gateway-side controller, an edge server, or a network planning platform, without requiring terminal nodes to participate in the computation. Nevertheless, the runtime, memory consumption, number of convergence iterations, and hardware resource requirements of the proposed algorithm have not yet been evaluated on practical LoRa gateways, embedded edge devices, or commercial servers. In addition, the current optimization objective mainly focuses on coverage probability maximization and does not explicitly incorporate relay installation cost, location adjustment cost, power supply conditions, maintenance cost, candidate site availability, or other practical deployment constraints. Future work should further evaluate the computational efficiency of the algorithm on practical deployment platforms. Deployment cost, energy constraints, site constraints, and operation and maintenance costs should also be incorporated into the relay placement optimization model as additional constraints or penalty terms to improve the practical applicability of the proposed method in real LoRa network deployments.

### 6.3. Discussion of Limitations

The proposed analytical framework is based on several simplifying assumptions, primarily Rayleigh small-scale fading and independent Bernoulli traffic activation. These assumptions enable the derivation of tractable coverage-probability expressions and allow the effects of path loss, thermal noise, random traffic activation, intra-SF interference and residual inter-SF interference on the coverage performance of two-hop LoRa networks to be explicitly characterized. Nevertheless, they may constrain the direct applicability of the model to practical LoRa deployments.

The Rayleigh fading model is primarily applicable to LoRa scenarios characterized by non-line-of-sight propagation, pronounced multipath scattering or the absence of a stable direct propagation path. In scenarios involving clear line-of-sight links, strong dominant components, terrain-induced shadowing or building blockage, the actual channel may not be adequately represented by the Rayleigh assumption. Therefore, the proposed model is more suitable for analyzing relative performance trends under different relay locations, traffic loads and interference conditions than for directly predicting the exact measured coverage probability in a specific deployment environment.

The independent Bernoulli traffic-activation assumption is used to describe the random activation states of potential interfering nodes within each observation cycle. This assumption is more suitable for LoRa sensor networks in which node reporting events are relatively dispersed, traffic arrivals are approximately independent, the network operates under a non-saturated traffic load and no strong synchronous triggering mechanism is present. Under conditions with a large number of nodes, a relatively stable traffic-activation probability, a known SF region partition and weak inter-node traffic correlation, the proposed model can provide reliable predictions of the average network coverage performance and can be used to compare the relative performance of different relay deployment schemes.

By contrast, in event-triggered monitoring, burst alarm reporting, disaster sensing or applications with strong spatial correlation, multiple nodes may be activated within a short time interval, leading to correlated traffic activation and bursty interference. In such cases, the independent Bernoulli assumption may underestimate the probability of clustered active interfering nodes and result in overly optimistic coverage-performance predictions. In addition, the proposed model does not explicitly consider the acknowledgment mechanism, receive windows, duty-cycle constraints, scheduling overhead, cooperative multi-gateway reception or hardware-implementation errors in the full LoRaWAN protocol stack. Therefore, for practical deployment scenarios involving strongly correlated traffic, complex blockage, multi-gateway cooperation or full LoRaWAN protocol constraints, the results of this study should be interpreted primarily as a theoretical benchmark and a reference for relative performance comparison.

In summary, the proposed analytical framework is primarily applicable to evaluating average coverage performance in LoRa networks, analyzing interference effects under random traffic activation, comparing the relative performance of different relay deployment schemes and supporting offline or semi-offline relay-placement planning. Future work will further incorporate Rician fading, shadow fading, terrain-induced blockage, correlated traffic-activation models and hardware testbed validation to improve the prediction accuracy and engineering applicability of the model in practical LoRa deployments.

## 7. Simulation Results and Analysis

Numerical simulations are conducted using MATLAB R2024a to validate the accuracy of the dual-hop LoRa coverage probability model and the effectiveness of the relay placement optimization method under random traffic activation. Monte Carlo simulations are performed to obtain statistical averages over random topology realizations. The simulations evaluate the effects of traffic activation intensity, relay placement strategy, optimization convergence behavior, path-loss exponent, reception threshold, and node density on the coverage probability. For comparison, several benchmark schemes are considered, including the direct-link baseline, the deterministic dual-hop baseline based on rounded active-node numbers, random relay deployment, fixed relay deployment, and the per-topology optimal upper bound. The direct-link baseline corresponds to the case in which the source node transmits data directly to the gateway without relay assistance. In contrast, the deterministic dual-hop baseline replaces the random traffic activation process with a deterministic approximation based on the rounded expected number of active nodes, and the corresponding dual-hop coverage probability is then evaluated. “Random-traffic-aware dual-hop” denotes a dual-hop transmission scheme in which the Bernoulli traffic-activation probability is explicitly incorporated into the interference model. “Random-traffic-aware cooperative coverage reference” denotes the theoretical reference performance obtained by treating the direct link and the dual-hop link as complementary transmission opportunities under the same random traffic-activation conditions. Table 3 lists the simulation settings for the main parameters.

**Figure 2:**
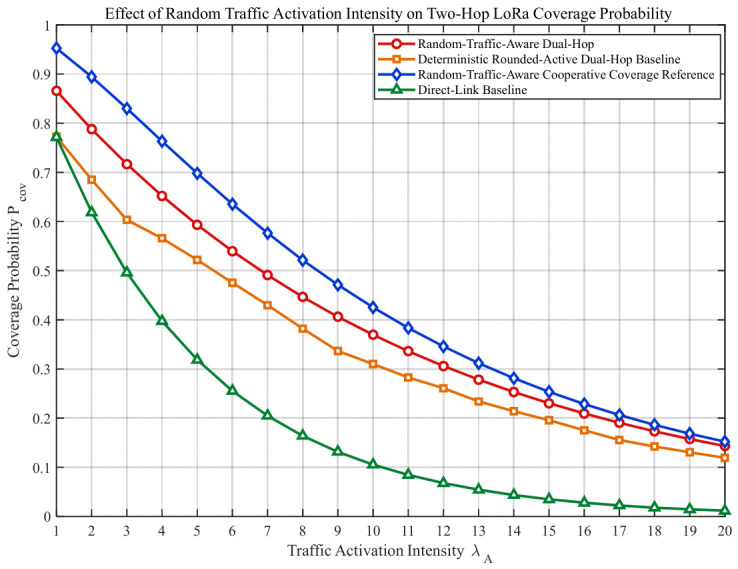
Effect of random traffic activation intensity on two-hop LoRa coverage probability.

Figure 2 illustrates the variation in the coverage probability $P_{cov}$ under different transmission schemes as the traffic-activation intensity coefficient $\lambda_{A}$ increases from 1 to 20. Overall, the four curves decrease monotonically as $\lambda_{A}$ increases. This indicates that a higher random traffic-activation intensity results in more active interfering nodes in the current observation cycle. Consequently, co-SF interference and residual inter-SF interference are jointly intensified, thereby reducing the successful transmission probability of the target link. An increase in the activation probability $p_{k}\left(\lambda_{A}\right)$ reduces the Laplace factor of each Bernoulli–Rayleigh interference term and consequently decreases the overall coverage probability. Among the considered schemes, the random-traffic-aware cooperative coverage reference consistently yields the highest coverage probability. The random-traffic-aware dual-hop scheme ranks second, followed by the deterministic dual-hop baseline based on rounded active-node numbers. The direct-link baseline exhibits the most pronounced degradation as the traffic-activation intensity increases.

The random-traffic-aware dual-hop curve remains higher than the deterministic dual-hop baseline based on rounded active-node numbers over the entire range. When $\lambda_{A}=5$, the coverage probability of the random-traffic-aware dual-hop scheme is approximately 0.56, whereas that of the deterministic dual-hop baseline is approximately 0.48, corresponding to an improvement of about 16.7%. When $\lambda_{A}=10$, the corresponding values are approximately 0.35 and 0.29, respectively, corresponding to an improvement of about 20.7%. These results indicate that modeling traffic activation as a Bernoulli random process captures the stochastic nature of practical LoRa uplink traffic more effectively than using a deterministic rounded number of active nodes. A closer examination of the cooperative coverage reference curve shows that it remains higher than both the standalone dual-hop scheme and the direct-link baseline over the entire range of $\lambda_{A}$. When $\lambda_{A}=5$, the cooperative coverage probability is approximately 0.66, which is about 17.9% higher than that of the random-traffic-aware dual-hop scheme at 0.56 and about 106.3% higher than that of the direct-link baseline at 0.32. When $\lambda_{A}=10$, the cooperative coverage probability is approximately 0.40 and still exceeds both the dual-hop and direct-link coverage probabilities. These results indicate that the direct link and the dual-hop link provide a certain degree of coverage complementarity. Under specific random topology and traffic-activation realizations, the direct link may still succeed, whereas the dual-hop link can provide additional reliability when the direct link is severely constrained by path loss. When $\lambda_{A}$ exceeds 15, the coverage probabilities of all schemes decrease markedly. The performance gaps among the cooperative coverage reference, the random-traffic-aware dual-hop scheme and the deterministic dual-hop baseline based on rounded active-node numbers also gradually narrow. This behavior occurs because a large number of potential interfering nodes are activated under high traffic-activation intensity, which substantially increases the aggregate interference intensity and renders both the dual-hop and direct links severely interference-limited.

**Figure 3:**
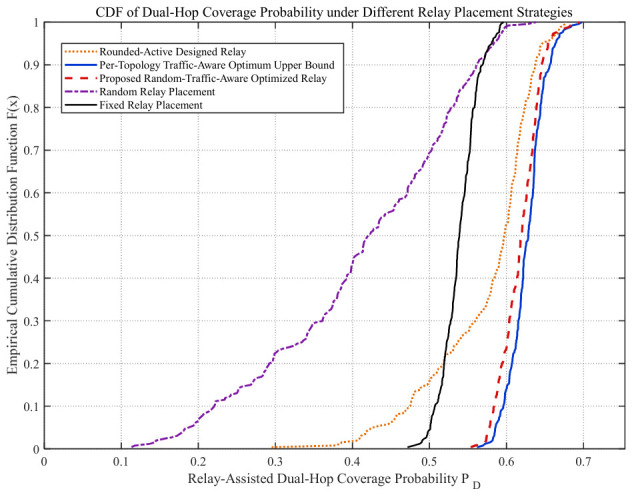
Empirical CDF of dual-hop coverage probability under different relay placement strategies.

Figure 3 presents the empirical cumulative distribution functions of the relay-assisted dual-hop coverage probability $P_{D}$ under different relay deployment strategies. The horizontal axis denotes the end-to-end dual-hop coverage probability, and the vertical axis denotes the empirical cumulative distribution function $F\left(x\right)$. For the CDF curves, a rightward shift indicates a higher dual-hop coverage probability at the same cumulative probability level and therefore better system coverage performance. In addition, a steeper curve reflects smaller performance fluctuations across different random topologies and greater deployment stability.

The overall distribution of the CDF curves indicates that the random-traffic-aware per-topology optimal upper bound and the proposed method are located on the right-hand side, suggesting that they yield higher dual-hop coverage probabilities than the other benchmark strategies. The random-traffic-aware per-topology optimal upper bound represents the case in which the relay position is re-optimized for each test topology and can therefore be regarded as an ideal performance upper bound. Although the proposed method uses a fixed optimized relay position obtained from the training topologies, its CDF curve is very close to that of the per-topology optimal upper bound. This demonstrates that the proposed random-traffic-aware relay optimization method exhibits strong generalization capability and deployment stability.

At the median point $F\left(x\right)=0.5$, the dual-hop coverage probabilities for random relay placement, fixed relay placement, the deterministic rounded-active-node relay design, the proposed method and the random-traffic-aware per-topology optimal upper bound are approximately 0.42, 0.54, 0.59, 0.62 and 0.63, respectively. This result indicates that directly incorporating the random traffic-activation probability into the relay-position optimization objective enables the optimized relay position to match the actual active-interference field more accurately and thereby improves the dual-hop coverage performance. The deterministic rounded-active-node relay design outperforms the fixed relay placement in the middle- and high-percentile regions, but its coverage probability in the low-percentile region is approximately 0.48, which is lower than the approximately 0.58 achieved by the proposed method. This indicates that although the deterministic rounded-active-node method improves relay deployment by using the rounded expected number of active nodes, it neglects the probabilistic structure of the active interferer set under Bernoulli random activation. Consequently, coverage degradation still occurs in some random topologies. In contrast, the proposed method directly formulates the optimization objective using the random traffic-activation probability and maintains higher coverage probabilities in the low-percentile, median and high-percentile regions, demonstrating stronger adaptability to random traffic fluctuations.

**Figure 4:**
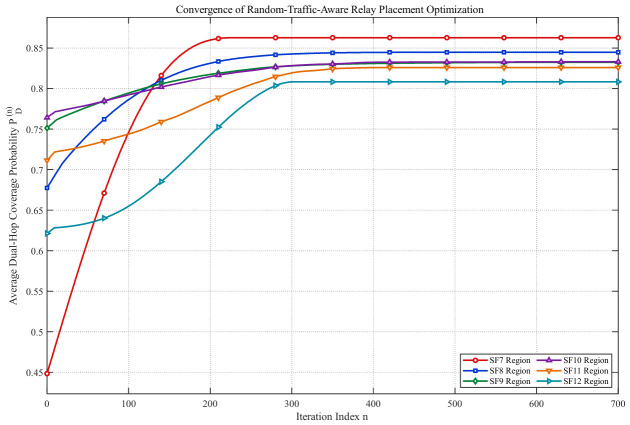
Convergence of the random-traffic-aware relay placement optimization algorithm.

Figure 4 shows the convergence curves of the random-traffic-aware relay-position optimization algorithm. The maximum number of iterations is set to 700, and the convergence behavior is evaluated across six candidate relay partitions associated with SF7–SF12. Overall, the coverage probability of each SF partition increases monotonically with the number of iterations and gradually converges to a stable value. This indicates that the adopted relay-position optimization algorithm progressively improves the end-to-end dual-hop coverage performance under random traffic activation, path loss and co-SF/inter-SF interference conditions. No noticeable oscillation or performance degradation is observed, demonstrating good numerical stability. Among the six partitions, the SF7, SF8, SF9 and SF12 partitions exhibit more rapid improvement during the early iterations, indicating that their initial relay positions have greater spatial adjustment potential toward their respective local optima. The SF10 and SF11 partitions exhibit more gradual improvement, suggesting that their coverage-probability surfaces are smoother or that their initial points are already close to high-coverage regions. The curves eventually approach stable values, indicating limited additional coverage gains from further iterations and effective convergence of the algorithm. Overall, the results confirm the effectiveness and convergence of the proposed random-traffic-aware relay-position optimization method. They show that appropriate relay-position adjustment can improve the average coverage probability of dual-hop LoRa networks under random traffic activation and provide useful guidance for selecting a more favorable relay deployment partition.

**Figure 5:**
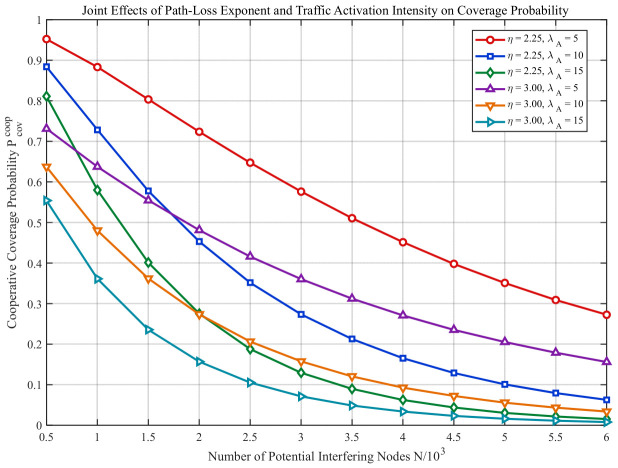
Impact of path loss exponent and traffic activation intensity on coverage probability.

Figure 5 examines the joint effects of the path-loss exponent η, the traffic-activation intensity $\lambda_{A}$ and the number of potential interfering nodes N on the cooperative coverage probability $P_{cop}^{coop}$. All curves decrease monotonically as N increases, indicating that a larger population of potential interferers leads to more active interfering nodes. As a result, the aggregate interference increases and the cooperative coverage performance degrades.

For a fixed path-loss exponent, a higher traffic-activation intensity leads to a more pronounced reduction in coverage probability. When η=2.25 and N=3000, the cooperative coverage probabilities for $\lambda_{A}=5$, 10 and 15 are approximately 0.58, 0.35 and 0.14, respectively. When N increases to 6000, the corresponding probabilities further decrease to approximately 0.27, 0.06 and 0.02. These results indicate that the traffic-activation intensity plays a critical role in determining the coverage performance of dense LoRa networks. For a given traffic-activation intensity, the coverage probability for η=2.25 is generally higher than that for η=3. For example, when $\lambda_{A}=5$ and N=3000, the cooperative coverage probability is approximately 0.58 for η=2.25 and approximately 0.37 for η=3. When N=6000, the corresponding probabilities decrease to approximately 0.27 and 0.16, respectively. This result indicates that in the edge-node dual-hop communication scenario considered in this study, the adverse effect of a larger path-loss exponent on the received power of the desired links outweighs the potential benefit from stronger attenuation of distant interference.

Overall, the cooperative coverage performance is jointly determined by the number of potential interfering nodes, the traffic-activation intensity and the path-loss exponent. Specifically, a larger N expands the potential interference field, a higher $\lambda_{A}$ increases the proportion of actually active interfering nodes and a larger η aggravates the path loss of the desired links. Therefore, in dense traffic scenarios, dual-hop cooperation alone is insufficient to fully offset the impact of high-load interference. Relay-position optimization and traffic-load control should be jointly considered to improve the coverage reliability of edge nodes.

**Figure 6:**
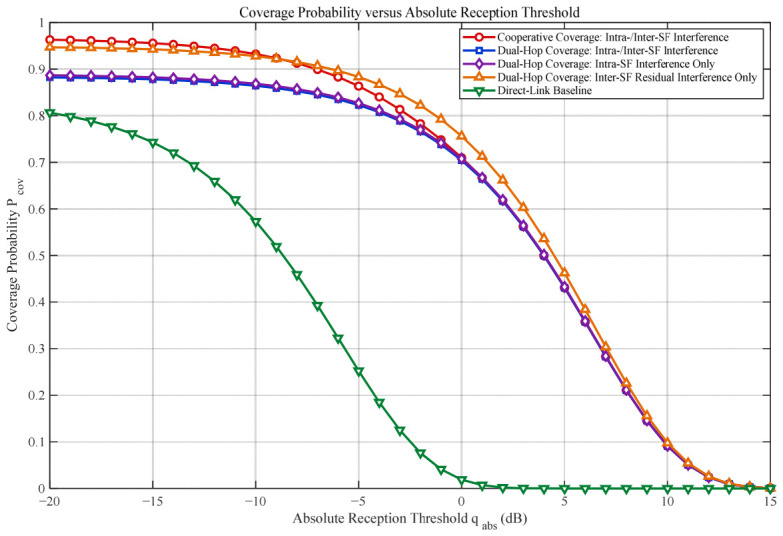
Coverage probability versus absolute receiving threshold.

Figure 6 examines the effect of the absolute reception threshold $q_{abs}$ on the coverage probabilities of different links. As $q_{abs}$ increases from −20 dB to 15 dB, the coverage probabilities of all schemes decrease markedly. This indicates that a higher reception threshold requires a larger channel gain for successful demodulation and strengthens the limiting effects of thermal noise and aggregate interference. In the low-threshold region, all schemes maintain relatively high coverage probabilities. However, when the threshold approaches 0 dB, the coverage probabilities decrease rapidly, suggesting that a high reception threshold substantially reduces the effective coverage range of LoRa links.

The cooperative coverage curve reflects the complementary gain between the direct link and the dual-hop link. The dual-hop coverage curve indicates that relay assistance effectively mitigates the path-loss limitation associated with direct communication between edge nodes and the gateway. In contrast, the direct-link baseline exhibits the fastest decline, indicating that long-distance direct links are the most sensitive to the reception threshold. The interference decomposition results show that the coverage probability remains relatively high when only residual inter-SF interference is considered. However, the coverage probability decreases markedly when co-SF interference is further included. This indicates that co-SF interference is the dominant factor limiting LoRa coverage performance, whereas residual inter-SF interference further aggravates the performance loss. Figure 6 demonstrates the joint effects of the reception threshold, co-SF/inter-SF interference and relay assistance on the system coverage probability. These results confirm that dual-hop relaying and refined co-SF/inter-SF interference modeling are effective in improving coverage performance under random traffic activation.

**Figure 7:**
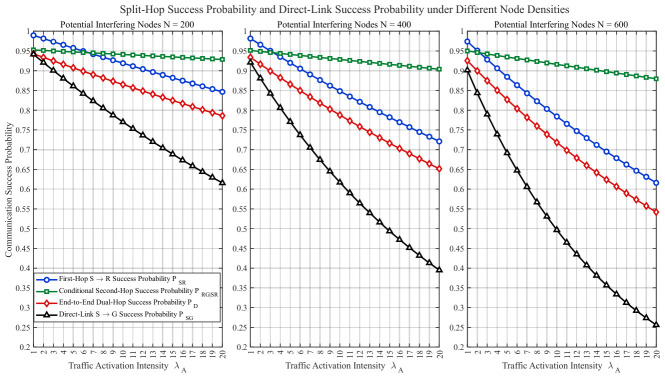
Hop-wise and direct-link success probabilities under different node densities.

Figure 7 illustrates the variations in the first-hop success probability, the conditional second-hop success probability, the end-to-end dual-hop success probability and the direct-link success probability with the traffic-activation intensity coefficient $\lambda_{A}$ under different potential-interferer densities. As $\lambda_{A}$ increases, more interfering nodes become active, and all success-probability curves exhibit corresponding downward trends. This indicates that an increased random traffic load progressively degrades link reliability. The comparison among the three node-density settings shows that coverage performance degrades more markedly as the node density increases. The direct-link curve is the most sensitive to node density and traffic-activation intensity and exhibits the fastest decline. This indicates that direct communication between edge source nodes and the gateway is jointly constrained by path loss and aggregate interference. Figure 7 shows that direct-link reliability becomes insufficient under high node density and heavy traffic load. In contrast, dual-hop relaying effectively improves the end-to-end communication success probability. These results demonstrate the effectiveness of hop-by-hop modeling and relay-assisted coverage optimization under random traffic activation.

## 8. Conclusions

A framework for dual-hop LoRa coverage probability analysis and relay-position optimization is developed to address the limited reliability of direct links for LoRa edge nodes under random traffic activation. Potential interfering nodes are partitioned into the SF7–SF12 regions according to their spatial locations. Cycle-level Bernoulli variables are then used to characterize the stochastic transition from potential interferers to active interfering nodes, thereby linking the traffic activation intensity, SF-specific activation probabilities and aggregate interference. The simulation results indicate that increases in traffic activation intensity, node density and the reception threshold reduce the coverage probability. Same-SF interference is the dominant performance-limiting factor, while residual inter-SF interference remains non-negligible under high-load conditions. Compared with the fixed-relay scheme, the random-relay scheme and the deterministic active-node approximation scheme, the proposed random-traffic-aware relay-position optimization method achieves more stable dual-hop coverage performance. Several limitations remain in the present study. First, the adopted channel model is mainly based on Rayleigh fading and does not fully account for line-of-sight components or terrain-induced blockage. The traffic model assumes independent Bernoulli activation and does not explicitly consider spatially distributed multi-gateway cooperative reporting scenarios. The cooperative coverage performance has been evaluated mainly through MATLAB simulations, and the proposed framework has not yet been validated on a full hardware testbed to assess the effects of ACK mechanisms, receive windows, duty-cycle constraints, scheduling overhead and dual-hop forwarding in a complete LoRaWAN protocol implementation. Future research will focus on multi-relay and multi-gateway deployments, hardware testbed validation, correlated traffic activation and constrained candidate relay locations. Overall, the proposed method provides a useful reference for improving edge-node communication quality and optimizing relay-assisted coverage in LoRa networks.

## Figures and Tables

**Table 1:** Key parameter symbols and definitions.

Symbol	Definition
$d_{A,B}$	Large-scale path loss of link A → B
$O_{A,B}$	Target SF index of link A → B
$a_{i}^{\left(q\right)}$	Activation state of the i-th node in cycle q
$p_{i}$	Traffic activation probability of the i-th node
$\lambda_{A}$	Traffic activation intensity coefficient
$p_{k}(\lambda_{A})$	Actual activation probability of the k-th SF region
$p_{A}^{k}$	Baseline activation probability of the k-th SF region
$L_{pac}$	Bit length of a single LoRa uplink packet
$T_{in}$	Used to define the cycle-level random traffic activation window
$\Omega_{A,B}$	Large-scale path-loss factor of link A → B
$K_{0}$	Reference path-loss constant
η	Path-loss exponent
$H_{A,B}$	Small-scale fading power gain of link A → B
$\Delta_{dB}\left(o,k\right)$	Element of the SF rejection-threshold matrix
$I_{B,same}\left(A\right)$	Aggregate same-SF interference
$I_{B,cross}\left(A\right)$	Residual inter-SF interference
$\alpha_{A,B,i}$	Equivalent interference coefficient of the i-th node for link A → B
${\tilde{\alpha }}_{A,B,i}$	Equivalent interference coefficient after mode selection
$J\left(x_{R}\right)$	Objective function for relay-position optimization
$x_{R}^{⋆}$	Optimal relay position
$\Omega_{R}$	Boundary and minimum-distance constraints for relay-node placement
$C_{S,R}$	First-hop success event
$C_{R,G}$	Second-hop success event
$P_{D}$	End-to-end coverage probability of the DF dual-hop link
D	End-to-end success event of the DF dual-hop link
$\delta_{o,k}$	Linear-domain SF interference weight
ς	Step-size reduction factor

**Table 2:** LoRa SF rejection-threshold matrix $\Delta_{dB}\left(o,k\right)$ in dB.

Target SF	Interfering SF7	Interfering SF8	Interfering SF9	Interfering SF10	Interfering SF11	Interfering SF12
SF7	1	−8	−9	−9	−9	−9
SF8	−11	1	−11	−12	−13	−13
SF9	−15	−13	1	−13	−14	−15
SF10	−19	−18	−17	1	−17	−18
SF11	−22	−22	−21	−20	1	−20
SF12	−25	−25	−25	−24	−23	1

**Table 3:** Main simulation parameters.

Parameter	System Parameters	Simulation Settings
System bandwidth	BW	250 kHz
Coding rate	CR	4/5
Carrier frequency	$f_{c}$	868 MHz
Network scale	$R_{0}$	6000 m
Path-loss exponent	η	2.70
Packet length	$L_{pac}$	80 bit
Observation cycle	$T_{in}$	60 s
Receiver sensitivity thresholds	$Q_{dB}$	$\left[-6,-9,-12,-15,-17.5,-20\right]$ dB
Transmit power	$P_{k}$	$\left[2,5,8,11,14,17\right]$ dBm
Small-scale fading	H	$\mathrm{Rayleigh},\;H∼Exp\left(1\right)$
Reference path-loss constant	$K_{0}$	31.21 dB
Noise figure	NF	6 dB

## Data Availability

The data supporting the findings of this study are available from the corresponding author upon reasonable request.

## Abbreviations

The following abbreviations are used in this manuscript:

LoRa	Long Range
LoRaWAN	Long Range Wide Area Network
LPWAN	Low-Power Wide-Area Network
IoT	Internet of Things
IIoT	Industrial Internet of Things
CSS	Chirp Spread Spectrum
SF	Spreading Factor
ToA	Time on Air
SNR	Signal-to-Noise Ratio
SINR	Signal-to-Interference-plus-Noise Ratio
DF	Decode-and-Forward
AF	Amplify-and-Forward
CDF	Cumulative Distribution Function
ACK	Acknowledgment
MAC	Medium Access Control

## Appendix A. Detailed Derivation of the Single-Hop Coverage Probability

For the target link A→B, the received power is given by $P_{A}H_{A,B}/\Omega_{A,B}$ as defined in the main text. The thermal noise power is denoted by $\sigma^{2}$, and the aggregate interference comprises same-SF interference and residual inter-SF interference. Accordingly, the coverage event can be written as

(A1) $$C_{A,B}=\left\{H_{A,B}\geq b_{A,B}+\sum_{i=1}^{N}a_{i}^{\left(q\right)}\alpha_{A,B,i}H_{i,B}\right\}$$

where $b_{A,B}$ denotes the normalized noise term and $\alpha_{A,B,i}$ denotes the equivalent interference coefficient of the i-th interfering node. This expression shows that the coverage event occurs when the Rayleigh channel gain exceeds the sum of the normalized noise term and the weighted interference terms.

Since the target-link channel gain satisfies $H_{A,B}∼Exp(1)$, conditioning on a given interference realization yields

(A2) $$P\left(C_{A,B}|\{a_{i}^{\left(q\right)},H_{i,B}{\}}_{i=1}^{N}\right)=exp\left[-b_{A,B}-\sum_{i=1}^{N}a_{i}^{\left(q\right)}\alpha_{A,B,i}H_{i,B}\right]$$

Equation (A2) follows from the tail probability of a unit-mean exponential distribution, i.e., Pr(H≥z)=exp(−z). By averaging over the random interference, the average single-hop coverage probability is obtained as

(A3) $$P_{A,B}=exp\left(-b_{A,B}\right)E\left[\prod_{i=1}^{N}exp\left(-a_{i}^{\left(q\right)}\alpha_{A,B,i}H_{i,B}\right)\right]$$

Assuming that the traffic activation states of the potential interfering nodes are independent of their small-scale fading gains and mutually independent across nodes, the expectation can be decomposed into a product of node-wise factors:

(A4) $$P_{A,B}=exp\left(-b_{A,B}\right)\prod_{i=1}^{N}E_{a_{i},H_{i,B}}\left[exp\left(-a_{i}^{\left(q\right)}\alpha_{A,B,i}H_{i,B}\right)\right]$$

For an individual interfering node, the corresponding factor reduces to one when $a_{i}^{\left(q\right)}=0$ because the node is inactive. When $a_{i}^{\left(q\right)}=1$, the expectation is taken over the Rayleigh fading gain of the interference link. Thus

(A5) $$E_{a_{i},H_{i,B}}\left[exp\left(-a_{i}^{\left(q\right)}\alpha_{A,B,i}H_{i,B}\right)\right]=\left(1-p_{i}\right)\cdot 1+p_{i}E_{H_{i,B}}\left[exp\left(-\alpha_{A,B,i}H_{i,B}\right)\right]$$

Since $H_{i,B}∼Exp(1)$, it follows that

(A6) $$E_{H_{i,B}}\left[exp\left(-\alpha_{A,B,i}H_{i,B}\right)\right]=\int_{0}^{\infty }e^{-\alpha_{A,B,i}h}e^{-h}\;dh=\frac{1}{1+\alpha_{A,B,i}}$$

Substituting (A6) into (A5) yields the Bernoulli–Rayleigh factor for an individual potential interfering node:

(A7) $$F_{A,B,i}=1-p_{i}+\frac{p_{i}}{1+\alpha_{A,B,i}}$$

By incorporating the interference-mode filtering defined in the main text, replacing $\alpha_{A,B,i}$ with ${\tilde{\alpha }}_{A,B,i}$ yields

(A8) $$F_{A,B,i}=1-p_{i}+\frac{p_{i}}{1+{\tilde{\alpha }}_{A,B,i}}$$

Substituting (A8) into (A4) gives the single-hop coverage probability:

(A9) $$P_{A,B}=exp\left(-b_{A,B}\right)\prod_{i=1}^{N}\left[1-p_{i}+\frac{p_{i}}{1+{\tilde{\alpha }}_{A,B,i}}\right]$$

Grouping the terms by SF region and setting $p_{i}=p_{k}\left(\lambda_{A}\right)$ for nodes in the k-th SF region yields Equation (26) in the main text:

(A10) $$P_{A,B}=exp\left(-b_{A,B}\right)\prod_{k=1}^{6}\prod_{x_{i}\in \Omega_{k}}\left[1-p_{k}\left(\lambda_{A}\right)+\frac{p_{k}\left(\lambda_{A}\right)}{1+{\tilde{\alpha }}_{A,B,i}}\right]$$

The derivation in Equation (A10) demonstrates how the random traffic activation probability, the weighted co-SF and inter-SF interference, and Rayleigh fading are jointly incorporated into the analytical expression for single-hop coverage probability.

$\alpha_{A,B,i}$ denotes the base equivalent interference coefficient of the i-th potential interfering node with respect to the target link A→B, before interference-mode filtering is applied. To incorporate different interference-mode settings into a unified expression, the mode-filtered coefficient ${\tilde{\alpha }}_{A,B,i}$ is introduced. Specifically, ${\tilde{\alpha }}_{A,B,i}$ is obtained by applying the interference-mode selection rule to $\alpha_{A,B,i}$. When both same-SF interference and inter-SF residual interference are considered, ${\tilde{\alpha }}_{A,B,i}=\alpha_{A,B,i}$. When only same-SF interference is considered, ${\tilde{\alpha }}_{A,B,i}=\alpha_{A,B,i}1\left(k_{i}=o_{A,B}\right)$. When only inter-SF residual interference is considered, ${\tilde{\alpha }}_{A,B,i}=\alpha_{A,B,i}1\left(k_{i}\neq o_{A,B}\right)$. Therefore, ${\tilde{\alpha }}_{A,B,i}$ is not an additional channel parameter but the mode-filtered form of $\alpha_{A,B,i}$.

Equation (A7) gives the Bernoulli–Rayleigh interference factor before interference-mode filtering is applied. To obtain the corresponding factor under a selected interference mode, $\alpha_{A,B,i}$ in Equation (A7) is replaced with the mode-filtered coefficient ${\tilde{\alpha }}_{A,B,i}$, which yields Equation (A8). If a potential interfering node is excluded by the selected interference mode, then ${\tilde{\alpha }}_{A,B,i}=0$, and the corresponding factor becomes $1-p_{i}+\left(p_{i}/1+0\right)=1$. This means that the node does not contribute effective interference under that mode. If the node satisfies the selected interference-mode condition, then ${\tilde{\alpha }}_{A,B,i}=\alpha_{A,B,i}$, and its interference contribution is retained. This explains the transition from Equation (A7) to Equation (A8).

## Appendix B. Derivation of the Bernoulli–Rayleigh Interference Laplace Factor

This appendix further clarifies the probabilistic interpretation of Equation (24). For any potential interfering node i, the activation indicator follows $a_{i}∼Bernoulli(p_{i})$, whereas the interference-channel gain follows $H_{i}∼Exp(1)$. Assuming that $a_{i}$ and $H_{i}$ are independent, the contribution of this node to the coverage probability is characterized by the following expectation:

(A11) $$F_{i}\left(s\right)=E_{a_{i},H_{i}}\left[exp\left(-sa_{i}H_{i}\right)\right]$$

where s denotes the normalized interference strength. Depending on the considered link, s is instantiated as ${\tilde{\alpha }}_{A,B,i}$, ${\tilde{\alpha }}_{S,R,i}$ or ${\tilde{\alpha }}_{R,G,i}$. By the law of total probability, we obtain

(A12) $$F_{i}\left(s\right)=P\left(a_{i}=0\right)\cdot 1+P\left(a_{i}=1\right)\cdot E_{H_{i}}\left[e^{-sH_{i}}\right]$$

Using $P\left(a_{i}=0\right)=1-p_{i}$, $P\left(a_{i}=1\right)=p_{i}$ and $H_{i}∼Exp\left(1\right)$, we obtain

(A13) $$F_{i}\left(s\right)=1-p_{i}+p_{i}\int_{0}^{\infty }e^{-sh}e^{-h}\;dh$$

Evaluating the integral yields

(A14) $$\int_{0}^{\infty }e^{-\left(1+s\right)h}\;dh=\frac{1}{1+s},\;\;s>-1$$

Therefore, the Laplace-transform factor for a single Bernoulli–Rayleigh interfering node is given by

(A15) $$F_{i}\left(s\right)=1-p_{i}+\frac{p_{i}}{1+s}$$

Setting $s={\tilde{\alpha }}_{A,B,i}$ yields the single-hop Laplace-transform factor used in the main text:

(A16) $$F_{A,B,i}=1-p_{i}+\frac{p_{i}}{1+{\tilde{\alpha }}_{A,B,i}}$$

Equation (A16) shows that increasing the traffic activation probability $p_{i}$ increases the likelihood that the i-th node contributes interference and therefore reduces the coverage probability. When $p_{i}=0$, the factor is equal to one, indicating that the node has no effect on coverage. When $p_{i}=1$, the factor reduces to the Rayleigh-only interference factor 1/(1+s).

## Appendix C. Derivation of the Joint Coverage Probability for DF Dual-Hop Transmission

This appendix derives Equation (29) in the main text. The end-to-end success event for DF dual-hop transmission is defined as

(A17) $$D=C_{S,R}\cap C_{R,G}$$

According to the coverage-decision model developed in the main text, the successful-reception condition for the first hop can be expressed as

(A18) $$H_{S,R}\geq b_{S,R}+\sum_{i=1}^{N}a_{i}{\tilde{\alpha }}_{S,R,i}H_{i,R}$$

The successful-reception condition for the second hop can be expressed as

(A19) $$H_{R,G}\geq b_{R,G}+\sum_{i=1}^{N}a_{i}{\tilde{\alpha }}_{R,G,i}H_{i,G}$$

The same activation variable $a_{i}$ in Equations (A18) and (A19) indicates that the traffic activation state of the i-th interfering node remains unchanged across the two hops within the same traffic-state window. Since $H_{S,R}$ and $H_{R,G}$ are independent and both follow exponential distributions with unit mean, we have

(A20) $$P\left(D|\{a_{i},H_{i,R},H_{i,G}\}\right)=exp\left[-b_{S,R}-\sum_{i=1}^{N}a_{i}{\tilde{\alpha }}_{S,R,i}H_{i,R}\right]exp\left[-b_{R,G}-\sum_{i=1}^{N}a_{i}{\tilde{\alpha }}_{R,G,i}H_{i,G}\right]$$

Rearranging the terms yields

(A21) $$P_{D}=exp\left(-b_{S,R}-b_{R,G}\right)\times E\left[\prod_{i=1}^{N}exp\left(-a_{i}{\tilde{\alpha }}_{S,R,i}H_{i,R}-a_{i}{\tilde{\alpha }}_{R,G,i}H_{i,G}\right)\right]$$

The expectation can be factorized into a product over individual nodes under the mutual independence assumption among the interfering nodes:

(A22) $$P_{D}=exp\left(-b_{S,R}-b_{R,G}\right)\prod_{i=1}^{N}G_{i}$$

where $G_{i}$ denotes the joint dual-hop factor of the i-th node. If this node is inactive, its contribution is one. If this node is active, it affects both hops simultaneously and thus yields

(A23) $$G_{i}=1-p_{i}+p_{i}E_{H_{i,R},H_{i,G}}\left[exp\left(-{\tilde{\alpha }}_{S,R,i}H_{i,R}\right)exp\left(-{\tilde{\alpha }}_{R,G,i}H_{i,G}\right)\right]$$

Since $H_{i,R}$ and $H_{i,G}$ are independent and both follow exponential distributions with unit mean, we have

(A24) $$E\left[e^{-{\tilde{\alpha }}_{S,R,i}H_{i,R}}\right]=\frac{1}{1+{\tilde{\alpha }}_{S,R,i}}\;,\;E\left[e^{-{\tilde{\alpha }}_{R,G,i}H_{i,G}}\right]=\frac{1}{1+{\tilde{\alpha }}_{R,G,i}}$$

Substituting Equation (A24) into Equation (A23) yields

(A25) $$G_{i}=1-p_{i}+\frac{p_{i}}{\left(1+{\tilde{\alpha }}_{S,R,i}\right)\left(1+{\tilde{\alpha }}_{R,G,i}\right)}$$

The joint coverage probability for DF dual-hop transmission is finally obtained as

(A26) $$P_{D}=exp\left(-b_{S,R}-b_{R,G}\right)\prod_{i=1}^{N}\left[1-p_{i}+\frac{p_{i}}{\left(1+{\tilde{\alpha }}_{S,R,i}\right)\left(1+{\tilde{\alpha }}_{R,G,i}\right)}\right]$$

## Appendix D. Derivation of the Cooperative Coverage Probability for Direct-Link and Dual-Hop Transmission

This appendix derives Equations (30)–(32) in the main text. The system-level coverage success event is defined as $C_{coop}=C_{S,G}\cup D$. By the probability formula for the union of events, the cooperative coverage probability is given by

(A27) $$P_{C}=P\left(C_{S,G}\right)+P\left(D\right)-P\left(C_{S,G}\cap D\right)$$

Let $P_{J}=P\left(C_{S,G}\cap D\right)$ denote the joint success probability. Substituting $D=C_{S,R}\cap C_{R,G}$ yields $P_{J}=P\left(C_{S,G}\cap C_{S,R}\cap C_{R,G}\right)$.

By writing the coverage decision condition for each of the three links and applying the tail probability of the exponential channel gain under Rayleigh fading, the conditional expression for the three-link joint coverage probability is obtained as

(A28) $$P_{J}=exp\left(-b_{S,G}-b_{S,R}-b_{R,G}\right)\times E\left[\prod_{i=1}^{N}exp\left(-a_{i}{\tilde{\alpha }}_{S,G,i}H_{i,G}-a_{i}{\tilde{\alpha }}_{S,R,i}H_{i,R}-a_{i}{\tilde{\alpha }}_{R,G,i}H_{i,G}^{\prime }\right)\right]$$

In Equation (A31), $H_{i,G}$, $H_{i,R}$ and $H_{i,G}^{\prime }$ denote the interference-link fading gains from the i-th interfering node to the direct-link receiver, the first-hop receiver, and the second-hop receiver, respectively. These gains are treated as mutually independent under the independent Rayleigh fading assumption across different links and transmission phases.

For a single interfering node, the corresponding joint factor across the three links is given by

(A29) $$H_{i}=1-p_{i}+\frac{p_{i}}{\left(1+{\tilde{\alpha }}_{S,G,i}\right)\left(1+{\tilde{\alpha }}_{S,R,i}\right)\left(1+{\tilde{\alpha }}_{R,G,i}\right)}$$

Thus, the three-link joint coverage probability is given by

(A30) $$P_{J}=exp\left(-b_{S,G}-b_{S,R}-b_{R,G}\right)\prod_{i=1}^{N}\left[1-p_{i}+\frac{p_{i}}{\left(1+{\tilde{\alpha }}_{S,G,i}\right)\left(1+{\tilde{\alpha }}_{S,R,i}\right)\left(1+{\tilde{\alpha }}_{R,G,i}\right)}\right]$$

Substituting Equation (A30) into Equation (A27) yields the cooperative coverage probability expression presented in the main text:

(A31) $$P_{C}=P_{S,G}+P_{D}-P_{J}$$
